# Origin and interpretation of cancer transcriptome profiling: the essential role of the stroma in determining prognosis and drug resistance

**DOI:** 10.15252/emmm.201505284

**Published:** 2015-08-03

**Authors:** Nicolas Goossens, Yujin Hoshida, Julio A Aguirre-Ghiso

**Affiliations:** 1 Division of Liver Diseases, Department of Medicine, Liver Cancer Program, Tisch Cancer Institute, Icahn School of Medicine at Mount SinaiNew York, NY, USA; 2 Division of Gastroenterology and Hepatology, Geneva University HospitalGeneva, Switzerland; 3 Division of Hematology and Oncology, Department of Medicine, Department of Otolaryngology, Department of Oncological Sciences, Tisch Cancer Institute, Icahn School of Medicine at Mount SinaiNew York, NY, USA

## Abstract

Mesenchymal gene expression in tumors has been implicated in cancer recurrence, metastasis, and poor prognosis of patients. The source of these mesenchymal signals has been mostly attributed to the epithelial-to-mesenchymal transition-like phenotype of epithelial tumor cells. However, recent evidence from colorectal and other cancer transcriptome studies clearly shows that the mesenchymal gene expression likely originates from stromal cells in and around the tumor and that this microenvironment specifically confers tumor aggressiveness. These findings highlight the need to move away from tumor-centric interpretations and to better establish the complementary role of the stromal microenvironment in fueling aggressive traits of cancer cells. This observation also suggests that future attempts at transcriptome profiling of whole tumor tissue must take into account the origin of mesenchymal gene expression profiles to better guide development of diagnostic and therapeutic strategies for cancer.

## A mesenchymal gene signature is commonly linked to poor epithelial cancer prognosis

Since the advent of genome-wide transcriptome profiling by DNA microarray, gene signatures of poor prognosis and chemoresistance in cancer tissue have been actively explored as a means to establish improved prognostic and diagnostic tools in the clinic, in addition to providing powerful discovery platforms. This has led to the gene signature-based molecular classification of cancers, which has been successfully implemented in clinical decision-making, for example in breast cancer care. Such gene signatures serve not only as predictive/prognostic biomarkers, but also as discovery tools for specific molecular deregulations that drive biologically and clinically aggressive tumor behavior, which are often shared across a range of cancer types. It has been noted that overexpression of mesenchymal genes (mesenchymal gene signature) is linked to poorer prognosis and the therapeutic resistance commonly arising in various cancer settings (Farmer *et al*, 2009). Mesenchymal gene activation was recurrently observed in a subset of colorectal cancers (CRC) in multiple independent transcriptome profiling studies, which led to the identification of a molecular CRC subclass robustly linked to lower histological differentiation, distinctly worse prognosis, and chemoresistance (De Sousa *et al*, 2013; Sadanandam *et al*, 2013). The gene signatures were determined in whole tumor tissue, and it was assumed that the mesenchymal gene expression originated from tumoral epithelial cells acquiring features of mesenchymal cells via epithelial-to-mesenchymal transition (EMT). EMT is a key mechanism in normal development, including gastrulation and organ morphogenesis, and also thought to be involved in cancer recurrence, metastasis, and drug resistance (Sosa *et al*, 2014). The molecular features of EMT (EMT-like features) are often associated with cancer stem cells (CSC), a tumor-initiating cell type expressing stem-cell markers (Sosa *et al*, 2014), and the formation of distant tumor metastasis by disseminating tumor cells (DTC) or circulating tumor cells (CTC) (Mitra *et al*, 2015). However, EMT does not define a clear univocal phenotype, but rather a continuum of plastic cell states such that EMT-like molecular features may be heterogeneously presented among cells within a tumor nodule (Nieto, 2013).

## The “poor-prognosis” mesenchymal signature is determined by the tumor stroma, not the epithelial tumor cells

**Figure 1:**
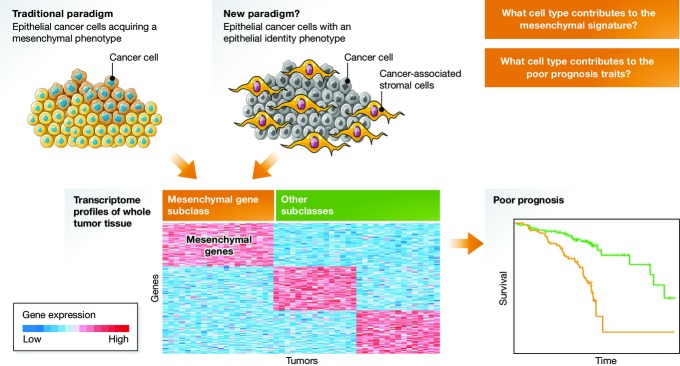
Origin of mesenchymal gene expression associated with poor prognosis in cancer Top left: Traditional paradigm, indicating the bulk of epithelial tumor cells (yellow and blue) that acquire a mesenchymal phenotype (e.g. EMT-like features) as the source of mesenchymal gene expression (bottom left). These features are undoubtedly associated with poor prognosis (bottom right). Top right: New paradigm, attributing the source of mesenchymal gene expression to stromal cells (orange) in the tumor nodule (gray). The EMT-like tumor lesions on the left certainly contain stromal cells as well, further complicating the identification of the source of the EMT traits even in more “mesenchymal” epithelial tumors. (Cell cartoons from www.servier.com.)

Recent follow-up studies of the CRC EMT-like transcriptome subclass unexpectedly uncovered that the source of mesenchymal gene expression is not the bulk of epithelial tumor cells, but stromal cells such as the cancer-associated fibroblasts within the tumor nodule (Calon *et al*, 2015; Isella *et al*, 2015). This finding was verified in patient-derived organoid and xenograft models, and transforming growth factor (TGF)-β signaling was identified as a key factor in the genesis of the poor-prognosis mesenchymal gene signature. These studies underscore that the transcriptome profile of a whole tumor nodule consists of mixed signals of molecular deregulation originating from cells of different types and origin in the nodule, and challenge our interpretation of the EMT program in the cancer cells (Fig1). To establish that the source of poor-prognosis-associated mesenchymal gene expression is a subpopulation of cells of mesenchymal lineage, and not epithelial tumor cells with EMT-like features such as CSCs, despite appearing self-evident, is a fundamental conceptual distinction. In some cases, tumor cells may be the source of the mesenchymal gene expression via trans-differentiation of CSCs into stromal cells as has been documented in glioblastoma stem-like cells (Wang *et al*, 2010), but the larger proportion of stromal cells recruited from the surrounding tissues may still significantly contribute to these signals. In addition, in a murine model of inflammation-induced gastric cancer, a significant proportion of intratumoral fibroblasts might derive from bone marrow mesenchymal stem cells (Quante *et al*, 2011).

Multiple cancer types display molecular subtypes with EMT-like features, but their association with prognosis is variable, suggesting that the nature of mesenchymal signals is different across tissue/cancer types (Tan *et al*, 2014). Nevertheless, these recent studies clearly highlight the need to better characterize the cell-type populations within a lesion to interpret the mesenchymal- or EMT-like molecular signatures. Specifically, cell type-specific deregulation of the mesenchymal/EMT markers should be determined to clarify their source; recently described technological advances, as described below, should help to achieve this goal.

The biological information conveyed by mesenchymal signatures must also be considered to clarify which role(s) they might play in the different steps of disease progression. Given that tumor-associated stromal cells do not appear to disseminate together with tumor cells to the site of distant metastasis and that metastatic lesions can take a long time to manifest, the stromal cells within the primary tumor might imprint DTCs with specific molecular programs that guide formation of metastatic foci. Elucidation of such mechanisms may be relevant to allow therapeutic targeting of DTCs and micro- and macro-metastases in secondary organs. On the other hand, stromal signals at the primary tumor site might, in some cases, bear little influence on metastasis features that eventually determine patient prognosis. In conclusion, there is clearly a need to characterize the target organ stroma with the same level of detail dedicated to the primary tumor stroma.

## Perspective

The prognostic relevance of mesenchymal gene expression in colorectal cancer tissue is indisputable and clinically relevant. The most straightforward way to detect mesenchymal cell components in the tumor may be by histopathology and imaging, although subjectivity may limit accurate quantitative assessment. Computer-assisted deconvolution of cell type-specific gene expression may further refine and improve the accuracy of detection. It is currently clinically impossible to determine the origin of the tumor stromal cells and track their fate; therefore, experimental systems that allow longitudinal monitoring of the mesenchymal/EMT markers at the single cell level will be needed as well as the development of technologies to verify the findings in human specimens, at least in a cell type-specific manner. Emerging technologies allowing single-cell mRNA tracking (Park *et al*, 2014), single-molecule microscopy, or single-cell sequencing may address these technical challenges. These new technologies may also help determine how the mesenchymal gene signatures present in primary tumors are carried over by CTCs and DTCs that colonize secondary target sites to form metastatic foci.

Understanding the source of the mesenchymal signal is important in designing therapeutic strategies to include targeting of the stromal component of cancer. In this respect, blocking the metastasis-promoting positive feedback loop between breast cancer cells and macrophages (Ojalvo *et al*, 2010) or heat-shock factor 1-mediated programs of non-cell-autonomous malignancy (Scherz-Shouval *et al*, 2014) may be good examples.

In conclusion, clarification of the origin and function of the stromal gene expression in cancer may provide novel strategies to target cancer recurrence, even when arising after long periods of remission (Sosa *et al*, 2014). Furthermore, characterization of stromal gene expression may serve as an additional layer of information, complementary to the cancer genome, to better inform treatment options toward the achievement of “precision medicine”.
